# Motor unit number estimates and neuromuscular transmission in the tibialis anterior of master athletes: evidence that athletic older people are not spared from age‐related motor unit remodeling

**DOI:** 10.14814/phy2.12987

**Published:** 2016-09-30

**Authors:** Mathew Piasecki, Alex Ireland, Jessica Coulson, Dan W. Stashuk, Andrew Hamilton‐Wright, Agnieszka Swiecicka, Martin K. Rutter, Jamie S. McPhee, David A. Jones

**Affiliations:** ^1^ School of Healthcare ScienceManchester Metropolitan UniversityManchesterUK; ^2^ Department of Systems Design EngineeringUniversity of WaterlooWaterlooOntarioCanada; ^3^ Mathematics and Computer ScienceMount Allison UniversitySackvilleNew BrunswickCanada; ^4^ Andrology Research UnitCardiovascular, Metabolic and Nutritional Sciences DomainFaculty of Biology, Medicine and HealthUniversity of ManchesterManchesterUK; ^5^ Manchester Diabetes CentreCentral Manchester University Hospitals NHS Foundation TrustManchester Academic Health Science CentreManchesterUK

**Keywords:** Aging, electromyography, master athletes, motor unit, skeletal muscle

## Abstract

Muscle motor unit numbers decrease markedly in old age, while remaining motor units are enlarged and can have reduced neuromuscular junction transmission stability. However, it is possible that regular intense physical activity throughout life can attenuate this remodeling. The aim of this study was to compare the number, size, and neuromuscular junction transmission stability of tibialis anterior (TA) motor units in healthy young and older men with those of exceptionally active master runners. The distribution of motor unit potential (MUP) size was determined from intramuscular electromyographic signals recorded in healthy male Young (mean ± SD, 26 ± 5 years), Old (71 ± 4 years) and Master Athletes (69 ± 3 years). Relative differences between groups in numbers of motor units was assessed using two methods, one comparing MUP size and muscle cross‐sectional area (CSA) determined with MRI, the other comparing surface recorded MUPs with maximal compound muscle action potentials and commonly known as a “motor unit number estimate (MUNE)”. Near fiber (NF) jiggle was measured to assess neuromuscular junction transmission stability. TA CSA did not differ between groups. MUNE values for the Old and Master Athletes were 45% and 40%, respectively, of the Young. Intramuscular MUPs of Old and Master Athletes were 43% and 56% larger than Young. NF jiggle was slightly higher in the Master Athletes, with no difference between Young and Old. These results show substantial and similar motor unit loss and remodeling in Master Athletes and Old individuals compared with Young, which suggests that lifelong training does not attenuate the age‐related loss of motor units.

## Introduction

Loss of muscle mass and strength are implicated in the general decline in mobility and the propensity to fall that are common features of old age. The muscle loss may be due, in part, to atrophy of muscle fibers but detailed studies of the vastus lateralis (VL) have shown the main cause to be a reduced number of fibers (Lexell et al. 1988). Muscle fiber loss is associated with death of spinal motor neurons and direct counts of motor neuron cell bodies in postmortem specimens of human lumbar spine show around 30% fewer neurons innervating the leg muscles of old compared with younger people (Kawamura et al. 1977; Tomlinson and Irving 1977). This is supported by motor unit number estimates (MUNE) derived from electromyographic (EMG) measurements of several limb muscles suggesting a 30–50% loss of motor units by the age of 70 years (Piasecki et al. 2015a). The denervated muscle fibers do not necessarily atrophy and disappear, but may be reinnervated by sprouting of nearby axons of surviving motor neurons resulting in larger motor units (Luff 1998). Thus, for muscles of the same size, larger motor units are indicative of a smaller total number of motor units.

Any intervention that could prevent or reduce the age‐related decline in muscle mass would be very valuable but, while there is considerable interest in addressing the changes in muscle protein turnover which occur with age that may be responsible for fiber atrophy (Sepulveda et al. 2015), there are no pharmacological or nutritional interventions known to affect the age‐related loss of motor units. However, there are suggestions that high levels of lifelong physical activity may protect against this loss. Power et al. (2010) reported higher MUNE values in the tibialis anterior (TA) of 10 master runners (mean age 64 years) compared with nonathletic people of similar age, although MUNE values for biceps brachii were similarly low in master runners and nonathletic old (Power et al. 2012). It was suggested that this was because the biceps, unlike the TA, were not loaded during running and did not receive the prolonged beneficial exercise stimulus (Power et al. 2012). However, continuing high levels of physical activity into the eighth and ninth decades does not appear to protect against motor unit loss. Comparing the MUNE data for master athletes and control subjects aged around 65 years in the study of Power et al. (2010) with those of around 80 years in a later study (Power et al. 2016), shows the master athletes deteriorating to a similar extent as the nonathletic old.

Aging is also associated with remodeling of the neuromuscular junction (Deschenes 2011; Gonzalez‐Freire et al. 2014), a process that may be influenced by activity. Recently master athletes were reported to have less transmission variability than nonathletic old (Power et al. 2016) as measured by the variation in shape across consecutive motor unit potentials (MUP), known as “jiggle” (Stålberg and Sonoo 1994).

While the suggestion that lifelong exercise can preserve motor units is attractive, the evidence is based on only one study of MUNE (Power et al. 2010) and one of jiggle (Power et al. 2016), which provide no information about muscle size or intramuscular MUPs. The aim of this study was to compare the muscle size, strength, estimates of motor unit number, size, and neuromuscular junction transmission stability in the TA of three groups: healthy young and older men together with exceptionally active master athletes. In line with the studies by Power et al. (2010, 2016), it was hypothesized that if high levels of lifelong exercise can preserve motor units, then the master athletes should have higher MUNE values, smaller MUP size, and lower jiggle than the nonathletic old, but be closer to the young adults in these respects.

## Methods

### Participants and ethical approval

The study was conducted in accordance with the *Declaration of Helsinki* and approved by the University Research Ethics Committee. Eighteen young men, 14 older men, and 13 male master athletes participated in the study and provided informed and written consent.

### Participant physical activity history

The Young and Old participants were recruited from the university population and the local community. All participants in the Young and Old groups were healthy and recreationally active. Master Athletes were recruited from participants in two national Masters Athletics competitions, and from an advertisement placed in a national athletics magazine. One of the 13 athletes ran in sprint events ≤400 m and the remaining 12 competed in events ≥3000 m. The master athletes were asked to estimate the number of hours per week they had devoted to athletic training throughout their lifetime. Up to the age of 18, and between ages 18 and 30 years, the median value was 3 h, and from 30 to 50 years, it increased to 6 h per week. Aged over 50 years, the median training hours remained at 6 per week. All master athletes were competing at the time of testing and had achieved the merit standards of the British Masters Athletics Federation (BMAF, 2015) in their respective distances and age groups at least once within the previous 2 years. The Master Athlete group included four currently ranked in the top three in Great Britain for their respective distances. The age‐graded performance (AGP) of an athlete is the approximate world‐record time for the athlete's age divided by the athlete's actual time. The mean AGP for the Master Athletes, expressed as a percentage, was 79 ± 6. Exclusion criteria were a recent history of bone fracture or neuromuscular, metabolic or cardiovascular disease.

### Anthropometric assessments

**Figure 1:**
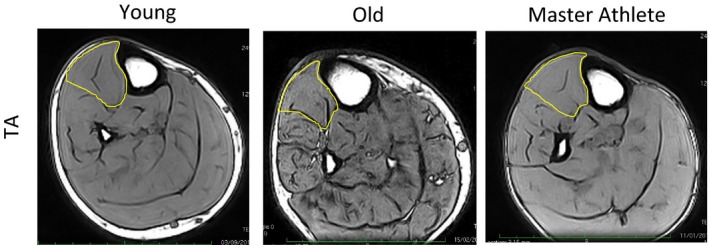
MRI images of the lower leg of Young, Old, and Master Athlete participants. The tibialis anterior (TA) muscles are outlined in yellow. These images were taken from the mid‐belly motor point of the TA.

The cross‐sectional area (CSA) of the TA at the level of the motor point (approximately mid‐muscle belly) was measured in the right leg with magnetic resonance imaging using a T1‐weighted turbo 3D sequence on a 0.25‐T G‐Scan with the participants lying supine (Esaote, Genoa, Italy). Contiguous transverse‐plane slices of 6 mm thickness were collected and typical images are shown in Figure 1. Images were exported and analyzed off‐line using Osirix imaging software (Osirix medical imaging, Osirix, Atlanta, GA) by tracing around the outer border of the muscle fascia (Maden‐Wilkinson et al. 2014). Body mass and height were measured using calibrated scales and stadiometry, and the body mass index (BMI) was calculated using them. Total body fat percentage was assessed by dual‐energy X‐ray absorptiometry (Lunar Prodigy Advance, version EnCore 10.50.086; GE Healthcare, Little Chalfont, UK) with the participant lying supine with legs and arms fully extended (McPhee et al. 2013).

### Strength assessments

The maximal isometric voluntary contraction (MVC) of the ankle dorsiflexors was measured with the participant sitting with the hips flexed at around 60° (lying supine being 0°), both legs fully extended and the right foot securely strapped into a custom‐built dynamometer (Jones et al. 2009). Participants were allowed to warm up and become accustomed to dorsiflexion contraction by performing a series of 10 brief, moderate intensity contractions over 2 min. MVC force was established as the best of three maximal efforts separated by around 40 sec rest intervals, but additional attempts were allowed if there was greater than 10% variation between the best two MVC efforts. Verbal encouragement and real‐time visual feedback on the computer screen were provided throughout.

### Identifying the motor point

The motor point was identified as the site that produced the largest twitch when using a very low stimulating current with a cathode probe (Medserve, Daventry, UK) and a self‐adhesive anode electrode (Dermatrode, Fermadomo, BR Nuland, the Netherlands) placed over the medial knee joint cleft. A constant current stimulator (DS7AH Digitimer, Welwyn Garden City, Hertfordshire, UK) was set at 400 V and 50 *μ*s pulse width. Current was varied to find the lowest level (typically around 8 mA) that induced a localized muscle twitch at the motor point with no response evident when applied elsewhere.

### Surface EMG signals

The active sEMG electrode (disposable self‐adhering Ag‐AgCl electrodes; 95 mm^2^_,_ Ambu Neuroline, Baltorpbakken, Ballerup, Denmark) was placed over the motor point and positioned to give the largest M‐wave and shortest rise‐time in response to stimulating the motor nerve (described below). The reference electrode was placed over the patella tendon and a common ground electrode over the patella, which served for both surface and iEMG measurements. Surface EMG signals were bandpass filtered between 5 Hz and 5 kHz via CED 1902 amplifiers (Cambridge Electronics Designs Ltd, Cambridge, UK). Signals were digitized with a CED Micro 1401 data acquisition unit (Cambridge Electronic Designs). The sEMG signals were sampled at 10 kHz.

### Compound muscle action potential

**Figure 2:**
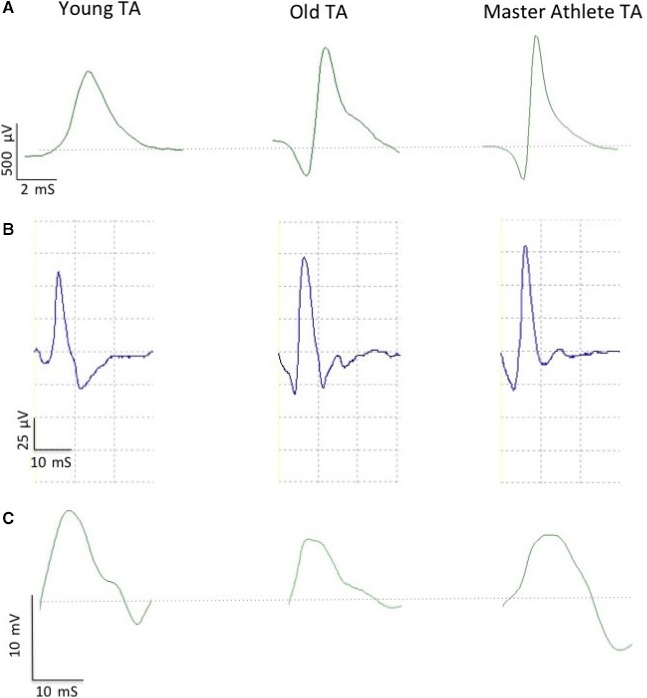
Example electromyographic data from tibialis anterior (TA) of Young, Old, and Master Athlete. (A) Individual intramuscular motor unit potential. (B) Ensemble‐averaged surface motor unit potential. (C) Compound muscle action potential. Dotted line in (A) and (C) indicates baseline (0.00 mV).

Compound muscle action potentials (CMAP) were evoked using a manually triggered stimulator (model DS7A; Digitimer). A bar electrode with the anode and cathode spaced 3 cm apart (Model MLADDF30; AD Instruments, Oxford, UK) was held over the common peroneal nerve around 5–10 mm distal of the fibular notch. For both muscles, the stimulator was set at 400 V, pulse width 50 *μ*s, and the current increased incrementally by 30 mA until the CMAP amplitude plateaued, generally between 100 and 150 mA. The current was then increased by 30 mA to ensure supramaximal stimulation. Typical CMAP traces are shown in Figure 2C.

### Intramuscular EMG signals

After determining the MVC and CMAP, a concentric needle electrode (Model N53153; Teca, Hawthorne, NY) was inserted around 1–1.5 cm into the TA immediately adjacent to the active sEMG electrode over the motor point. The intramuscular signals (iEMG) were bandpass filtered from 10 Hz to 10 kHz and sampled at 25 kHz. The force and EMG signals were displayed in real‐time using Spike 2 (v8.01; Cambridge Electronic Designs Ltd) and data were stored for off‐line analysis.

### Recording from individual motor units during voluntary contractions

The participant performed a low force voluntary contraction while the needle position was adjusted to obtain intramuscular motor unit potentials (iMUPs) with peak second derivative values >5 kV/sec^2^. The participant then performed a voluntary contraction lasting 12–15 sec, keeping as close as possible to a target set at 25% MVC with real‐time visual feedback. The needle electrode was then repositioned by combinations of rotating the bevel 180° and withdrawing it by around 3–5 mm. The procedure of needle positioning, voluntary contraction, and signal recording was repeated until a minimum of six recordings from spatially distinct regions at varying depths had been obtained. The participant rested for 15–30 sec between contractions.

### EMG signal analysis and motor unit number estimates

**Figure 3:**
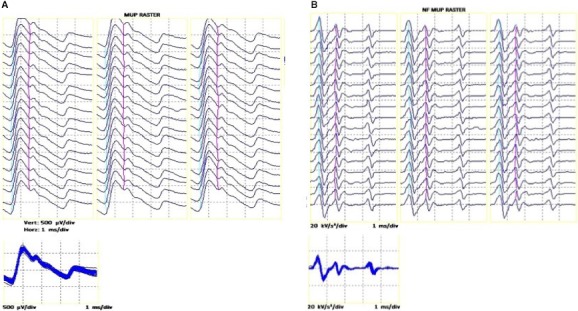
Repeated firing of a single intramuscular motor unit potential (A) and its corresponding near fiber (NF) motor unit potential (B), shown consecutively as a raster plot and also overlaid in the subplots. All data are taken from a master athlete.

The procedures for recording and analyzing individual MUPs and calculating near fiber (NF) and MUNE values have been described in detail (Hourigan et al. 2015; Piasecki et al. 2015b; Power et al. 2016). Briefly, intramuscular and surface EMG signals were analyzed using decomposition‐based quantitative electromyography (DQEMG) software (Stashuk 1999; Boe et al. 2005, 2006). MUNE values were obtained using spike‐triggered ensemble‐averaging, in which MUP occurrence times identified from the iEMG signal were used to trigger sEMG signal epochs which were then ensemble‐averaged allowing surface motor unit potentials (sMUPs) to be extracted (Brown et al. 1988). Figure 2A shows an individual iMUP, such as used for spike‐triggered ensemble‐averaging. The sMUPs, as shown in Figure 2B, were onset aligned to create an ensemble‐averaged mean sMUP. The negative peak amplitude of the averaged sMUP was then divided into the negative peak amplitude of the electrically evoked maximal CMAP (Fig. 2C) to obtain a MUNE value. A NF MUP was created by applying a high‐pass filter to each iMUP; this effectively reduces the recording volume of the iEMG electrode by emphasizing contributions from fibers close to the electrode detection surface relative to more distant fibers and creating a iMUP essentially generated by the NF. Raster plots of a typical MUP and corresponding NF iMUP are shown in Figure 3. The MU firing rate was identified from consecutive occurrences of individual iMUPs. NF count is a feature of a NF iMUP related to fiber density and obtained by counting the number of significant and symmetrical negative peaks.

Data analysis was carried out with the analyst blinded to the participant's age and training status.

Estimates related to the numbers of motor units were also made assuming that iMUP area is proportional to the CSA of the fibers of the generating motor unit. Dividing the CSA of a muscle by the mean iMUP area (cm^2^/mV/msec) provides an estimate related to the total number of MUs within that cross section of muscle and is referred to as the intramuscular MUNE (iMUNE; Piasecki et al. 2015b).

### Statistical analysis

A univariate analysis of variance was used to identify differences in participant and neuromuscular characteristics between groups, shown in Tables 1 and 2. When a significant main effect was observed, a post hoc test with Bonferroni correction was used to identify where significant differences existed. Data are presented as mean (SD) or, if not normally distributed, as median (IQR). Statistical significance was accepted at *P* < 0.05. Statistical analysis was performed using SPSS Version 21 (SPSS, Chicago, IL).

**Table 1:** Participant characteristics

	Young *n* = 18	Old *n* = 14	Master Athletes *n* = 13
Age (years)	26 (4)	71 (4)***	69 (3)***
Height (cm)	176 (5)	171 (7)**	174 (6)
Weight (kg)	77.7 (10.8)	73.4 (9.1)	69.3 (7.1)*
BMI (kg/m^2^)	24.5 (3.8)	25.3 (3.9)	22.9 (2.9)
Body fat (%)	14.1 (7.3)	25.1 (7.2)***	14.8 (5.8)†††
Total lean mass (kg)	64.9 (7.2)	52.8 (4.7)**	56.5 (5.4)**
TA CSA (cm^2^)	9.6 (1.9)	8.5 (1.8)	8.9 (1.1)
Dorsiflexion MVC (N)	371 (144)	245 (58)**	290 (52)*

BMI, body mass index; CSA, cross‐sectional area; MVC, maximum voluntary contraction force.

Significant differences compared to Young identified by post hoc analysis of the results are shown as: *P < 0.05; **P < 0.01; ***P < 0.0005. Significant differences between Old and Master Athletes are shown as ^†††^ *P* < 0.0005.

**Table 2:** Neuromuscular characteristics of tibialis anterior

	Young (*n* = 18)	Old (*n* = 14)	Master Athletes (*n* = 13)
iMUP area (*μ*V ms)	1004 (313)	1434 (421)**	1575 (538)**
Firing rate (Hz)	12.4 (1.5)	10.8 (1.3)**	11.0 (1.6)**
NF Jiggle (%)	26.7 (4.3)	27.5 (2.5)	30.4 (4.4)*
NF Count	1.55 (0.47)	1.91 (0.66)	1.98 (0.61)
NF Area (kV/s^2^)	3.63 (1.49)	5.66 (2.79)*	7.79 (3.93)***
NF MUP duration (ms)	2.07 (0.48)	3.20 (0.86)***	3.68 (1.11)***
sMUP Amp (*μ*V)	52.7 (41.1–76.2)	75.4 (50–92.3)	63.2 (51.9–90.1)
CMAP Amp (*μ*V)	11,294 (3978)	7461 (3217)**	6205 (2216)***

iMUP, intramuscular motor unit potential; NF, near fiber; sMUP, surface motor unit potential; CMAP, compound muscle action potential. Data are shown as mean (SD), or if not normally distributed as median (IQR). Significant differences compared to Young identified by post hoc analysis of the results are shown as: *P < 0.05; **P < 0.01; ***P < 0.0005.

## Results

Participant characteristics are shown in Table 1. The Old were of a similar body mass and BMI to the Young but had a higher total body fat while the Master Athletes were the lightest group with significantly less body fat and lower BMI than the Old. Both older groups had a lower total lean mass than the young.

Muscle CSA of the TA did not differ significantly between Young, Old, and Master Athlete groups, although the Old and Master Athletes had lower MVC force than the Young.

### Surface EMG signals

**Figure 4:**
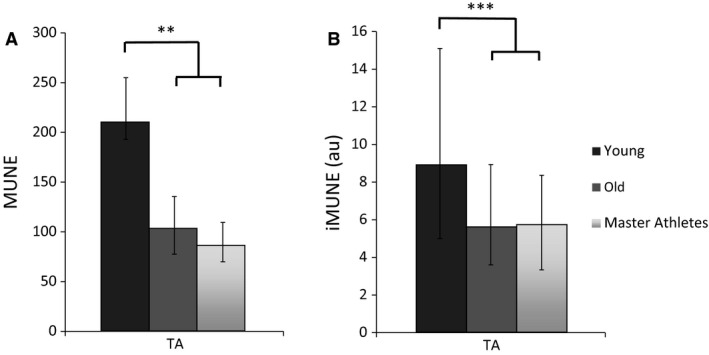
Values for motor unit number estimates in the tibialis anterior from Young, Old, and Master Athletes. (A) MUNE values calculated from the CMAP and sMUP amplitude. (B) iMUNE was calculated from muscle CSA the iMUP area. Data are shown as medians and IQR. There were no differences between Old and Master Athletes. MUNE, motor unit number estimate; au, arbitrary units; CMAP, compound muscle action potential; sMUP, surface motor unit potential; iMUP, intramuscular motor unit potential; CSA, cross‐sectional area. Significant differences between groups are shown as **P < 0.01 ***P < 0.0005.

The CMAP of the TA was larger in the Young compared to Old and Master Athletes but there was no significant difference between Old and Master Athletes. Surface MUP size did not differ between the three groups, so the MUNE value, calculated in the same way as in other studies of motor unit characteristics of athletic older people (Power et al. 2010, 2016), was greatest in Young, with no difference between Old and Master Athletes (Fig. 4).

### Intramuscular EMG signals

The mean iMUP areas were 43% and 56% larger for the Old and Master Athletes, respectively, compared with the Young, with no significant differences between Old and Master Athletes (Table 2).

**Figure 5:**
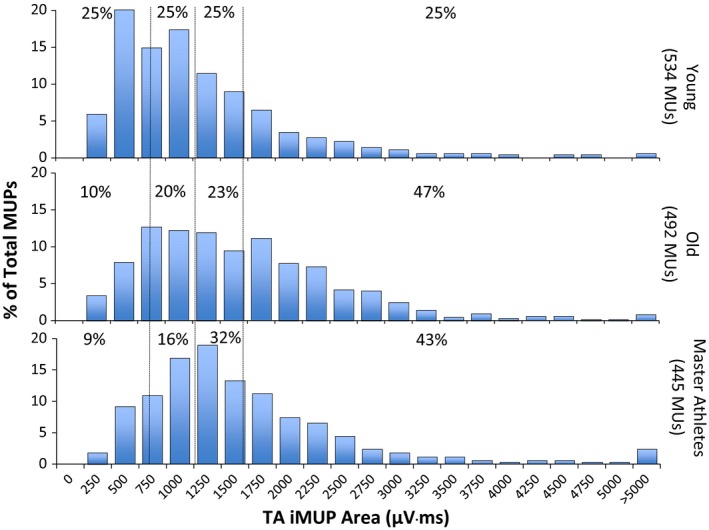
Distribution of intramuscular motor unit potential (iMUP) areas of tibialis anterior (TA) recorded during dorsiflexion at 25% maximum voluntary contraction force. Dotted vertical lines indicate quartile ranges for the Young and percentage values show the proportion of motor units within each Young quartile.

When all iMUPs were pooled by group, there was a rightward shift in iMUP area of the Old and Master Athletes compared with the Young (Fig. 5), the proportions of iMUPs falling in the lowest quartile of the Young distribution of size were 10% and 9% for Old and Master Athletes, respectively, while nearly half the iMUPs of Old and Master Athletes were of a size (47% and 43% for, respectively) that corresponded to the upper quartile of the Young distribution.

The mean firing rate was lower in the Old and Masters Athletes when compared to the Young, with no difference between the Old and Master Athletes (Table 2).

The NF Jiggle was higher in the Master Athletes compared to the Young, with no difference between Master Athletes and Old. There was no significant difference in NF Count between the three groups, although there was a trend for this to be higher in Master Athletes and Old compared with Young. The NF Area and Duration were significantly higher in the Old and Master Athletes than the Young, with no difference between the older groups (Table 2).

Using the mean iMUP area and muscle CSA to calculate iMUNE showed that the Old and the Master Athletes had 37% and 36% lower values, respectively, than the Young, with no difference between Old and Master Athletes (Fig. 4).

## Discussion

There are many benefits of regular physical activity for the middle aged and elderly (McPhee et al. 2016), and the idea that neuromuscular structure and function can be preserved into old age by maintaining regular and intense exercise is appealing. However, our main findings indicate that motor unit remodeling occurs to a similar extent in those who have trained and competed to a high level throughout their lives as with average healthy active older men.

### Participants, muscle mass, and function

The master athletes were all talented runners who had been training and competing at a high level for most of their adult life. The master athletes were lighter than the participants in the Old group, mainly due to their lower body fat content which is indicative of good metabolic and cardiovascular health.

There were no significant differences in CSA of the TA between the two older groups and the younger participants (Table 1), but the maximal dorsiflexion force was lower in the Old and the Master Athletes compared with Young. Consequently, the specific forces (MVC normalized to CSA) were 38, 31, and 33 N/cm^2^ for Young, Old, and Master Athletes, respectively. This difference may be due to a combination of reduced voluntary activation and increased noncontractile material in the older TA (Kent‐Braun et al. 2000).

### Motor unit number estimates

It is not possible to determine absolute numbers of motor units in large human muscles using EMG techniques, but the two methods used here provide estimates that allow relative differences between groups to be assessed. MUNE values calculated in the conventional way, as CMAP divided by sMUP (Brown et al. 1988), refer to a volume of muscle “seen” by the surface electrodes and while this may be the whole contractile mass for small muscles of the hand or foot, this is clearly not the case for larger muscles, including TA. In this respect, it is notable that mean MUNE values in healthy young participants are similar for a range of muscles of very different sizes; TA: 150 ± 43 (McNeil et al. 2005), thenar: 228 ± 93, biceps brachii: 113 ± 40, extensor digitorum brevis: 131 ± 45, vastus medialis: 229 ± 108 (Galea et al. 1991) and the soleus: 458 ± 151 (Dalton et al. 2008). We have previously reported median MUNE values in the much larger VL as 111 (96–156) (Piasecki et al. 2015b). MUNE values will tend to be biased by motor units that are closer to the skin surface due to signal attenuation of sMUPs from deeper units (Muceli et al. 2015). The conventional MUNE also suffers from some uncertainty as to whether the surface signals are contaminated by electrical signals from nearby muscles or attenuated by skin and subcutaneous tissue, so it is important to view the MUNE results alongside other indicators of neuromuscular structure and function.

The data presented in Table 2 and Figure 5 clearly show that Old and Master Athletes had larger iMUPs than Young, there being a shift in the distribution of iMUP size toward larger units recruited to produce the same relative force (25% MVC) (Fig. 5). The size of an iMUP is largely influenced by fibers within 2 mm of the intramuscular electrode detection surface (Nandedkar et al. 1988), and thus will reflect the number of electrically active fibers within that volume. Reinnervation is expected to increase the number of fibers within a motor unit and to do so largely within the territory of that unit, thereby increasing fiber density and the number of fibers contributing to an iMUP and thus generating larger potentials. In these circumstances, it is evident that for two muscles of the same size, the one with the larger motor units, as indicated by larger iMUPs, will have the smaller number of motor units. The TA of the Young, Old, and Master Athletes were all of similar size, while the iMUPs were larger for the two older groups. It follows, therefore, that the older participants had fewer motor units than the Young, with very little difference between Old and Master Athletes, as indicated by the iMUNE values shown in Figure 4. The slightly lower values of specific force of the older participants compared with the Young suggests the area of functional muscle may be smaller than the anatomical CSA used to calculate the iMUNE, perhaps due to more noncontractile material. This would mean that the iMUNE values of the older participants may be overestimated, so the real difference between Young, on the one hand, and Old and Master Athletes, on the other, would be even greater than shown here.

While both methods of estimating the relative differences in motor unit numbers between participant groups have some uncertainty, the fact that both show the same changes add confidence to the conclusion that master athletes are not protected against the age‐related loss of MUs that we and others have documented (Piasecki et al. 2015a).

The conclusion that master athletes are not protected from the neuromuscular changes associated with aging is not what was hypothesized on the basis of the conclusions of Power and colleagues (Power et al. 2010) who suggested that lifelong exercise preserves TA motor unit numbers. This discrepancy cannot be explained in terms of different methodology. Our TA MUNE values calculated using negative peak amplitude of CMAP and mean sMUP, the same method used by Power and colleagues, gave median values of 220, 100, and 90, respectively, for Young, Old, and Master Athletes which compare with mean values of 150, 91, and 150 reported by Power et al. (2010). The values for Young and Old are probably in the same range, but the difference lies in the values for the master athletes/runners. The master runners reported by Power and colleagues were 64 years old while in this study, the master athletes were 69 years old and, although there may be an accelerated decline of TA motor unit numbers with increasing older age (McNeil et al. 2005), the 5 years age difference between the two studies seems an unlikely explanation for the differences. Based on the mean race times of the athletes in Power's study, the mean AGP was around 73%, suggesting that the athletes in the two studies were of a similar standard. Consequently, it is not obvious why our results differ from those of Power et al. (2010), however, the CMAP and mean sMUP do require further comment. Power and colleagues found no difference between the three groups (young, old, and masters runners) in either of the two measurements, the difference in MUNE was driven by the slightly, but not significantly, larger mean sMUP in the old. The ensemble‐averaged sMUPs require a level of subjective operator input in the analytical process, whereas the CMAPs are a more robust measure. Given that in Power's study the data from the Young and Old were first published (and possibly collected) in 2005, several years before those of the athletes (McNeil et al. 2005), this variability in analysis of sMUPs may help to explain the discrepancy.

The values for NF jiggle of between 26% and 30% indicating neuromuscular junction transmission stability (Table 2) are similar to the values of 26–36% previously reported in this muscle for similar age groups (Hourigan et al. 2015), but are around 40% lower than values recently reported in master athletes (Power et al. 2016). Although the difference in fiber density, as measured by the NF count, was not significant in our study, there was a tendency for both older groups to have higher values than the Young. This was also true of the NF area and duration, although here the differences between young and older subjects were statistically significant. This is consistent with both older groups having larger and more fiber dense MUs, and further supports the notion that the Old are no different to the Master Athletes with respect of their motor unit structure and function. The reduced firing rate in both older groups is consistent with previous reports in this muscle (Klass et al. 2008) and may reflect the fact that the low/moderate threshold motor units (with lower firing rates) were enlarged (Fig. 5) and therefore able to produce sufficient force to meet the requirement to contract at 25% without recruiting higher threshold units with higher firing rates (Ling et al. 2009). It is known that older muscles tend to be slower than those of the young (Lexell 1995) and this is consistent with the enlargement of slower MUs at the expense of faster units.

Although the response to aging differs across muscles (Pannerec et al. 2016), McNeil et al. (2005) and Zampieri et al. (2015) both point out that the process of reinnervation compensates for the loss of motor units with age, acting to preserve the number of muscle fibers and thus muscle size and strength. The increase in iMUP and NF MUP size in the older participants shown here is an indication that reinnervation has compensated for the decrease in motor unit number and allowed muscle size to remain similar in Young and Old albeit, possibly, with more noncontractile material in the older muscles.

It may appear surprising that despite their sporting achievements, the master athletes’ motor units appear to be little different to those of age‐matched “normal” subjects. A full discussion of the factors determining sporting achievement is beyond the scope of this report, but two factors undoubtedly play a role in the superior performance of the athletes, one being their power to weight ratio, the other being their probable greater cardiovascular capacity.

### Limitations

All of the data presented here were collected during contractions at 25% of MVC and on the basis of the size order of MU recruitment (Henneman et al. 1965), it is likely that we have been sampling a range of smaller motor units. There are practical limitations to using higher force contractions, but it would be interesting to know if the enlargement of motor units reported here is also seen with units recruited at high force. While active during running, the TA is not the principle muscle generating the power and it would be useful to extend these observations to larger proximal muscles to determine whether lifelong exercise can protect these muscles from neuromuscular decline. This comparison of groups was a cross‐sectional study and longitudinal data are needed to further understand the rate of MU remodeling.

## Conclusion

There is a substantial body of evidence for distal muscle groups that the number of motor units, and by implication motor neurons, decrease with age. There is no doubt that physical activity is important for cardiovascular health and lifelong high levels of activity may also maintain muscle quality in various ways (Pollock et al. 2015; Zampieri et al. 2015). However, the data presented here demonstrate no substantial differences between healthy older individuals and master athletes, either in terms of muscle size and strength or motor unit numbers and structure, indicating that, contrary to expectation, master athletes are not protected from age‐related motor unit remodeling.

## Conflict of Interest

The authors have no conflicts of interest to declare.
